# Exploiting heterogeneous environments: does photosynthetic acclimation optimize carbon gain in fluctuating light?

**DOI:** 10.1093/jxb/erv055

**Published:** 2015-03-18

**Authors:** Renata Retkute, Stephanie E. Smith-Unna, Robert W. Smith, Alexandra J. Burgess, Oliver E. Jensen, Giles N. Johnson, Simon P. Preston, Erik H. Murchie

**Affiliations:** ^1^Division of Plant and Crop Sciences, School of Biosciences, University of Nottingham, Sutton Bonington Campus, LE12 5RD, UK; ^2^School of Mathematical Sciences, University of Nottingham, University Park, Nottingham NG7 2RD, UK; ^3^Sainsbury Laboratory, University of Cambridge, Bateman Street, Cambridge CB2 1LR, UK; ^4^Department of Plant Sciences, University of Cambridge, Downing Street, Cambridge CB2 3EA, UK; ^5^Systems and Synthetic Biology, Wageningen UR, Building 316, Dreijenplein 10, 6703HB Wageningen, Netherlands; ^6^School of Mathematics, University of Manchester, Oxford Road, Manchester M13 9PL, UK; ^7^Faculty of Life Sciences, University of Manchester, Michael Smith Building, Oxford Road, Manchester M13 9PT, UK

## Abstract

Plants have evolved complex mechanisms to balance the efficient use of absorbed light energy in photosynthesis with the capacity to use that energy in assimilation, so avoiding potential damage from excess light. This is particularly important under natural light, which can vary according to weather, solar movement and canopy movement. Photosynthetic acclimation is the means by which plants alter their leaf composition and structure over time to enhance photosynthetic efficiency and productivity. However there is no empirical or theoretical basis for understanding how leaves track historic light levels to determine acclimation status, or whether they do this accurately. We hypothesized that in fluctuating light (varying in both intensity and frequency), the light-response characteristics of a leaf should adjust (dynamically acclimate) to maximize daily carbon gain. Using a framework of mathematical modelling based on light-response curves, we have analysed carbon-gain dynamics under various light patterns. The objective was to develop new tools to quantify the precision with which photosynthesis acclimates according to the environment in which plants exist and to test this tool on existing data. We found an inverse relationship between the optimal maximum photosynthetic capacity and the frequency of low to high light transitions. Using experimental data from the literature we were able to show that the observed patterns for acclimation were consistent with a strategy towards maximizing daily carbon gain. Refinement of the model will further determine the precision of acclimation.

## Highlight

We propose a new mathematical model to empirically determine how successful plants are at maximizing daily carbon gain in fluctuating light according to dynamic acclimatory processes.

## Introduction

Light is one of the most variable resources for plants and is capable of changing by several orders of magnitude within fractions of a second. Solar movement, climate, clouds, canopy movement in the wind and canopy architecture can combine to produce a complex pattern of light in time and space. This has profound consequences for photosynthetic carbon assimilation in leaves, which can be slow to respond to changes in light. Light can rapidly shift from being limiting for photosynthesis to high levels that are sufficient to saturate the photosynthetic apparatus. Over the short term (seconds and minutes), the mechanisms that plants use to deal with these changes are relatively well understood: it is possible to invoke enzyme activation states, metabolite concentrations and the state of energization of the thylakoid membrane as a ‘memory’ of short-term past light history (Horton and Ruban, 2005; Murchie *et al.*, 2009; Garcia-Plazaola *et al.*, 2012). Short-term responses are regulated by processes such as phosphorylation of thylakoid components, allosteric regulation of enzymes and the physical state of the thylakoid (Tikkanen et al., 2010, 2012; Ruban *et al.*, 2012). Two examples of processes on such short timescales are photosynthetic induction—the delay in the rise in carbon assimilation immediately following a light increase (Pearcy *et al.*, 1997)—and thylakoid photoprotective processes, which result in a decline in quantum efficiency of photosynthesis as a response to excess light (Demmig-Adams and Adams, 1992; Murchie and Niyogi, 2011).

Longer-term responses, which occur over the timescale of days in response to changes in environmental conditions, are termed acclimation and are characterized by changes in leaf phenotype. Acclimation describes the alterations in quantity and stoichiometry of photosynthetic components—including Rubisco, cytochrome-b/f complexes, light harvesting complexes, ATPase and enzymes involved in carbohydrate synthesis—resulting in long-term changes to leaf properties such as photosynthetic capacity, dark respiration and the light compensation point (Bjorkman, 1981; Anderson *et al.*, 1995; Murchie and Horton, 1997, 1998; Walters *et al.*, 1999; Yano and Terashima, 2001; Walters, 2005; Athanasiou *et al.*, 2010). One can consider ‘sun’ and ‘shade’ leaf physiology as two extreme states of acclimation (Björkman, 1981; Murchie and Horton, 1997) and a scale of response between them is not necessarily linear (Bailey *et al.*, 2001). Exploration of the adaptive significance of acclimation under complex light patterns has however been little studied but is key to understanding the limits placed on plants in natural environments.

Two types of acclimation can be distinguished: the first refers to responses during leaf development and plastid biogenesis that determine cell numbers and size and leaf shape, that are largely irreversible (Weston *et al.*, 2000; Murchie *et al.*, 2005); the second type, here termed dynamic acclimation, is defined as the reversible changes that can occur in mature tissues in response to changes in the environment (Walters and Horton, 1994). The extent of the propensity to acclimate will depend on the plant’s genotype, which will, to a greater or lesser extent, match the environment to which it is adapted through evolution. Species from different ecological niches show differing abilities to acclimate (Anderson *et al.*, 1995; Murchie and Horton, 1997, 1998).

**Fig. 1.**
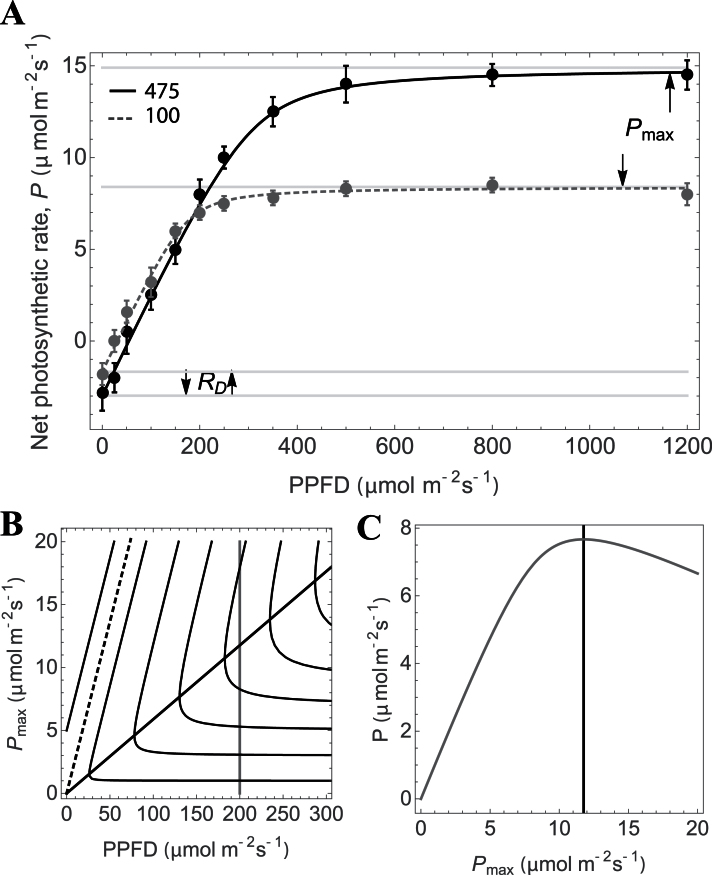
Net photosynthetic rate as a function of PPFD (light intensity *L*) and $P_{max}$. (A) Experimental data (Yin and Johnson, 2000) and fitted Eq (1). for *A. thaliana* grown under 475 μmol m^–2^ s^–1^ (black) and 100 μmol m^–2^ s^–1^ (grey) PPFDs. Data are mean ±se of 5−12 measurements. The light compensation point is where the curves cross the horizontal axis *P*=0. (B) The light-response surface: contours of constant net photosynthetic rate *P* are plotted in the positive quadrant of the $\left(L,P_{max}\right)$-plane. The dotted line indicates the light compensation point along which *P*=0 and the solid diagonal line is the locus of points for which *P* is maximized for fixed *L*. (C) *P* as a function of $P_{max}$ for a fixed *L*, corresponding to the vertical (grey) line in (B).

The acclimation state of a leaf can be readily defined in terms of its light response curve for photosynthesis. In the absence of light, the net rate of CO_2_ exchange will be negative and correspond to a dark respiration rate *R*_D_. With increasing amounts of light, the rate of photosynthesis, measured as the rate of CO_2_ uptake from the atmosphere, will increase, until a saturation point is reached. Experimentally, such responses are typically measured over a period of minutes as a light-response curve and can be modelled in C_3_ leaves using a non-rectangular hyperbola (Fig. 1A; see also Leverenz *et al.*, 1992; Ogren, 1993; Sharkey *et al.*, 2007) to relate net photosynthetic rate, *P* (photosynthetic CO_2_ uptake minus respiration rate) to photosynthetic photon flux density (PPFD), *L*. This curve is a useful reflection of the leaf’s current acclimation state and can be used to calculate its productivity. The slope of the light-response curve at *L*=0 describes the maximum efficiency with which light can be converted into fixed carbon. This is called the maximum quantum yield, *ϕ*. The net photosynthesis rate, *P,* rises until it reaches a maximum, $P_{max}$. The dark respiration rate *R*_D_ is the net rate of CO_2_ exchange in darkness (i.e. at *L*=0, where the curve meets the vertical axis). The value of *L* at which the curve crosses the horizontal axis (i.e. where the respiration rate equals the photosynthesis rate) is termed the light compensation point, where the PPFD takes the value *L*_c._

The non-rectangular hyperbola depends on parameters $P_{max}$, *R*_*D*_, *ϕ* and a convexity parameter, *θ*, which enable it to model C_3_ leaves, whether acclimated to low or to high light intensities. The shape of this curve will depend on the light absorption properties of the leaf (chlorophyll content, leaf thickness, etc.) and the relative concentrations of the different structures (proteins, cofactors) involved in assimilating the light energy (Adamson *et al.*, 1991; Chow *et al.*, 1991; Murchie and Horton, 1997). Despite the variation seen between and within species there are conserved trends that are useful for acclimation modelling approaches. The maximum quantum yield is unaffected by (non-stressful) growth conditions (Long and Drake, 1991). The leaf absorptance is unlikely to be substantially altered during dynamic acclimation (Pearcy and Sims, 1994). The rate of dark respiration *R*_D_ is known to vary depending on acclimation state, with low-light-acclimated leaves having lower *R*_D_ than high-light-acclimated leaves. *R*_D_ can be treated as being dependent on $P_{max}$according to the relationship *R*_D_=−α$P_{max}$, where α is assumed to be constant (Givnish, 1988; Niinemets and Tenhunen, 1997). Furthermore, in this paper we consider experimental conditions where the basic photosynthetic responses (maximum quantum yield, *ϕ*, and convexity, *θ*) for a given species are known and therefore we assume that a leaf’s acclimation state can be characterized using the value of $P_{max}$.

To account for the change in incident light, leaves presumably set their acclimation state based on a combination of current environmental signals and accumulated information from the past. When plants are transferred from low to high light, they typically acclimate to increase their maximum photosynthetic capacity ($P_{max}$), i.e. the light-saturated rate of photosynthesis. This process takes place over a period of 5–10 days, depending on species (Athanasiou *et al.*, 2010). Transfer from high to low light results in the opposite response, i.e. reducing $P_{max}$ (Walters, 2005). Dynamic acclimation is, at least to some extent, mechanistically different from developmental acclimation (Murchie and Horton, 1997; Athanasiou *et al.*, 2010). However, little is known about the way in which light signals are integrated through time to drive the acclimation response.

Optimal dynamic acclimation would track environmental conditions in real time, and match maximum photosynthetic capacity to the light level that the leaf directly experiences. However, as discussed above, acclimation is not an instantaneous process, and there is a time lag before the leaf fully responds to changes (Walters and Horton, 1994; Athanasiou *et al.*, 2010). The lag for increasing $P_{max}$ is thought to be longer than that for decreasing $P_{max}$, reflecting the fact that more proteins must be synthesized and maintained. Hence, the plant must invest carbon, nitrogen and other resources in order to sustain a higher photosynthetic capacity (Athanasiou *et al.*, 2010).

Mathematical models have been proposed to describe the response of plant photosynthetic processes to changes in external light conditions. These models have addressed the behaviour of key biochemical processes and plant physiology under variable light (Gross, 1982; Kirschbaum et al., 1988, 1997; Pearcy, 1990; Pearcy *et al.*, 1997), and changes in the dynamics of photosynthetic machinery due to environmental changes (Stegemann et al., 1999; Ebenhoeh *et al.*, 2011; Zaks *et al.*, 2012), to activation of enzymes and sucrose synthesis (Zhu *et al.*, 2013), and the role of crop canopy architecture on canopy photosynthesis (Song *et al.*, 2013; see Porcar-Castell and Palmroth, 2012 for a review of modelling photosynthesis under temporal variation in sunfleck activity). However, all of these models focus on time scales of seconds to minutes and all assume that the photosynthetic apparatus of the system modelled is constant.

It is often assumed that acclimation involves a strategy of optimisation geared toward maximum carbon gain in a given environment (Pons, 2012) but here we argue that our understanding is incomplete for complex light patterns. There are few empirical experiments in the literature that have explored how changes in light pattern influence the changes in $P_{max}$ (Chabot *et al.*, 1979; Watling *et al.*, 1997) and even fewer that have utilized light response curves (Yin and Johnson, 2000). Two of the available mechanisms discussed in the literature involve peak PPFD and integrated PPFD (Niinemets and Anten, 2009). No statistically significant differences in $P_{max}$ were found between plants grown under either constant or fluctuating light of the same integrated PPFD (Watling *et al.*, 1997). Extensive study under conditions of either constant integrated PPFD but variable peak PPFD, or constant peak PPFD but variable integrated PPFD, concluded that the integrated PPFD was a stimulus for photosynthetic acclimation to light (Chabot *et al.*, 1979). However later work noted that photosynthetic capacity changed in response to growth in fluctuating light patterns under the same integrated and peak PPFD, but varying duration of the high and low light period (Yin and Johnson, 2000). Therefore, the strategies that plants use are not completely understood and future studies should move beyond the concept of integrated versus peak PPFD.

In this study, we use mathematical modelling to investigate the optimal acclimation state for leaves that are subjected to a light pattern that varies. We propose a new approach that can be used to empirically determine how successful plants are at optimizing carbon gain in such conditions. We do not attempt to model how acclimation state changes with time, but our aim is instead to determine the efficiency of different fixed acclimation states for given light patterns.

## Materials and methods

### Theoretical framework

The net photosynthetic rate, *P*, as a function of PPFD, *L*, and maximum photosynthetic capacity, $P_{max}$, can be described by different mathematical formulas, for details see Supplementary Data S1B. In this study we use a non-rectangular hyperbola model proposed by Prioul and Chartier (1977) see Fig. 1A and Eq. (1).

$$\begin{array}{c} P\left(L,P_{max}\right)=\frac{\phi L+\left(1+\alpha \right)P_{max}-\sqrt{{\left(\phi L+\left(1+\alpha \right)P_{max}\right)}^{2}-4\theta \phi L\left(1+\alpha \right)P_{max}}}{2\theta }\\ -\alpha P_{max} \end{array}$$ (1)

Here *L* is the PPFD incident on a leaf (μmol m^–2^ s^–1^), ϕ is the maximum quantum yield, α corresponds to the fraction of the maximum photosynthetic capacity used for dark respiration, and the parameter θ determines the curvature of the light-response curve.

We define the daily carbon gain, *C* (mol m^–2^), in a fluctuating or constant environment as the integrated carbon over the time period *t* ϵ [0,*T*]:

$$C\left(L\left(t\right),P_{max}\right)=\overset{T}{\underset{0}{\int }}P\left(L\left(t\right),P_{max}\right)dt$$ (2)

In this study we have sought to predict a maximum photosynthetic capacity, $P_{max}^{opt}$, as the $P_{max}$ which represents maximum daily carbon gain for a given light pattern. We compared $P_{max}^{opt}$ with ${\bar{P}}_{max}$, which is defined as the $P_{max}$ at which the maximum daily carbon gain would be attained if the variable light pattern, *L(t*), were replaced by its average $\bar{L}$ over the time *T*.

The rate of photosynthesis at any instant is also determined by the state of induction of photosynthesis, which is a complex condition that represents the overall activation state of enzymes and electron carriers, pool sizes of photosynthetic metabolites and stomatal conductance (Gross, 1982; Kuppers and Pfiz, 2009). Induction state will determine the time taken to reach a steady state following an increase in light intensity.

Experimental data show that responses of photosynthesis to increases in irradiance are not instantaneous (Pearcy *et al.*, 1997). However, the available data is too limited for us to incorporate and parameterize accurately within our own model e.g. using an induction model such as that of Pearcy *et al.* (1997). Instead, as a simple way to capture ‘fading memory’ of the recent light pattern, we introduce a time-weighted average for the light:

$${\overset{}{L}}_{\tau }\left(t\right)=\frac{1}{\tau }\overset{t}{\underset{-\infty }{\int }}L\left(t'\right)e^{-\left(t-t^{'}\right)/\tau }dt'$$ (3)

Here we have used an exponentially decaying weight. This represents the concept that the leaf response to the previous light pattern is more strongly dominated by recent events. Thus for τ=0 the time-weighted averaged light pattern corresponds to its instantaneous value, whereas for τ>0, the time-weighted averaged light pattern relaxes over a timescale τ following a sudden change in *L*(*t*).

### Experimental data

In Yin and Johnson (2000), plants of *Arabidopsis thaliana* were grown for 4−6 weeks at a light intensity of 100 μmol m^–2^ s^–1^, then transferred to either a light environment that was constant during the photoperiod or an environment in which the light fluctuated between periods of low light intensity (100 μmol m^–2^ s^–1^) and high light intensity (475 μmol m^–2^ s^–1^) lasting 15min, 1h or 3h, for 7 d. The integrated PPFD for all fluctuating light patterns was 12.42mol m^–2^ d^–1^. As described by Yin and Johnson (2000), light response curves for oxygen evolution in leaf discs were taken in saturated CO_2_ (5%) at 20°C on leaf discs from dark-adapted leaves and therefore it is likely that light was the dominant limiting factor for photosynthesis in this experiment.

### Model parameterisation

Parameters were estimated from light response curves of *A. thaliana* grown under constant light conditions at 100 μmol m^–2^ s^–1^ and 475 μmol m^–2^ s^–1^ with a 12h photoperiod (Yin and Johnson, 2000). The non-rectangular hyperbola in Eq. (1) was fitted to the means of 5−12 measurements using a least-squares method. We inferred the following values: α=0.2, ϕ=0.055 and θ=0.96. Experimental data together with the fitted light response curves are shown in Fig. 1A. These values are comparable with other experimental studies: ϕ=0.043 for *A. thaliana* grown in controlled environment chambers with a 12h photoperiod at a PPFD of 250 μmol m^–2^ s^–1^ (Donahue *et al.*, 1997); and α=0.15 was found in Niinemets and Tenhunen (1997). All model analysis and model validation is done using these fitted parameter values.

As the same parameter values fitted both data sets (i.e. at 100 μmol m^–2^ s^–1^ and 475 μmol m^–2^ s^–1^), this suggests that photosynthetic acclimation to different growth conditions can be described using changes in $P_{max}$.

We calculated the time-weighted average of a given light pattern according to Eq. (3) with τ from 0.1h to 1h and calculated daily carbon gain using Eq. (2) for $P_{max}$ values from 0 to 80 μmol m^–2^ s^–1^ with step 0.01. We assigned $P_{max}^{opt}$ as a value that gives the highest daily carbon gain. To determine the best fit for τ we calculated a mean squared error between predicted and experimentally measured light response curves for plants grown under 6h switching period. This gave a value of τ=0.3h. Table 1 gives the list of symbols and parameter values.

**Table 1.** Symbol definitions

Symbol	Definition	Values/units
*k*	Fraction of time period spend under *L*_-_	[0,1]
*C*	Daily carbon gain	mol m^–2^
*L*	Instantaneous photosynthetic photon flux density (PPFD)	μmol m^–2^s^–1^
$\bar{L}$	Average of *L(t*) over the day	μmol m^–2^s^–1^
*L*c	Light compensation point	μmol m^–2^s^–1^
*L*_-_	Lower PPFD for two-level fluctuating light	100 μmol m^–2^s^–1^
*L*_+_	Higher PPFD for two-level fluctuating light	475 μmol m^–2^s^–1^
$L_{\tau }\left(t\right)$	Time-weighted average *L(t*) calculated for a given τ	μmol m^–2^s^–1^
*P*	Net photosynthetic rate	μmol m^–2^s^–1^
$P_{max}$	Maximum photosynthetic capacity	μmol m^–2^s^–1^
$P_{max}^{opt}$	Predicted $P_{max}$ for a given *L(t*) over a day	μmol m^–2^s^–1^
${\bar{P}}_{max}$	Predicted $P_{max}$ for $\bar{L}$	μmol m^–2^s^–1^
*R*_D_	Dark respiration rate	μmol m^–2^s^–1^
*S*	Switching period	h
*T*	Length of day	24h
α	Fraction of the maximum photosynthetic capacity used for dark respiration	0.2
θ	Convexity of light response curve	0.96
τ	Scale of a time weighted averaging	h
*ϕ*	Maximum quantum yield	0.055

## Results

### Quasi-steady net photosynthetic rate

First we look at a quasi-steady state, where leaves are subjected to a given ‘constant’ light intensity. Under such conditions, we model the relationship between net photosynthetic rate, *P* (*L*, $P_{max}$), maximum photosynthetic capacity, $P_{max},$ and the instantaneous PPFD, *L*, without considering the effect of photosynthetic induction.

The contours of constant *P* in the positive quadrant of the (*L*, $P_{max}$)-plane represent what can be termed the *light-response surface*. Fig. 1B shows contours for Eq. (1) for both varying $P_{max}$ and *L*. Traversing the surface horizontally at a fixed value of $P_{max}$ gives a light response curve similar to ones shown in Fig. 1A. If instead one follows the light-response surface for a fixed light PPFD (the grey line in Fig. 1B at *L=*200 μmol m^–2^ s^–1^) this will give *P* as a function of $P_{max}$. Fig. 1C shows that there is a value of $P_{max}$ that maximizes *P*; in this case it is 11.8 μmol m^–2^ s^–1^. This is a hypothetical example to help us illustrate a mechanism behind photosynthetic acclimation.

Acclimation is a long-term process in which we assume maximum photosynthetic capacity is adjusted to a particular light intensity, i.e. if PPFD is set to any fixed value *L,* acclimation involves moving vertically in Fig. 1B until the value of $P_{max}$ maximizes the net photosynthetic rate, *P*. For any *L* there is a well-defined $P_{max}$ that maximizes *P* (Fig. 1C); this corresponds to a point at which a contour of constant *P* in the (*L*, $P_{max}$)-plane is vertical, as indicated by the black diagonal line in Fig. 1B. Under higher light conditions, the $P_{max}$ that maximizes *P* for a given *L* is larger (moving along the black diagonal line in Fig. 1B).

#### Light pattern: alternation between two light levels

**Fig. 2.**
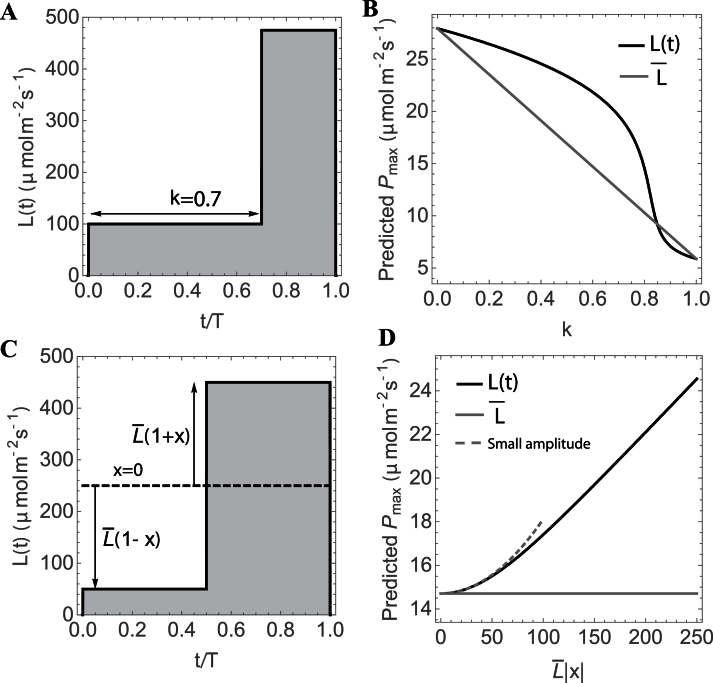
Photosynthetic acclimation under alternation between two light levels. (A) Fluctuating light pattern for *k*=0.7; (B) predicted $P_{max}$ as a function of low light duration, *k*; for *L*_-_=100 μmol m^–2^s^–1^ and *L*_+_=475 μmol m^–2^s^–1^ (C) Fluctuating light pattern for *k=0.5* and varying low/high PPFDs; (D) predicted $P_{max}$ as a function of the amplitude of fluctuations. Light intensity fluctuates between $\bar{L}\left(1-x\right)$ and $\bar{L}\left(1+x\right)$, where $\bar{L}$=212.5μmol m^–2^ s^–1^. In (B) and (D), the grey line corresponds to an averaged light intensity and the black line to the fluctuating light. In (D), the dashed grey line gives a small-amplitude approximation (see Supplementary Data S1).

Suppose that light fluctuates between two different intensities, such that, for given time, *t*, PPFD equals either *L*_-_ or *L*_+_, where *L*_-_≤ *L*_+._ In the time period 0≤*t*≤*T*, let the total time for which *L*(*t*)=*L*_-_ be *kT* and the total time for which *L*(*t*)=*L*_+_ be (1-*k*)*T*, where 0≤*k*≤1. The light pattern with *k=0.*7, *L*_-_=100 μmol m^–2^ s^–1^ and *L*_+_=475 μmol m^–2^ s^–1^ is shown in Fig. 2A.

Figure 2B shows how the $P_{max}^{opt}$ under fluctuating light depends on the value of *k*. The $P_{max}^{opt}$ under the average light intensity, $\bar{L}=kL_{-}+\left(1-k\right)L_{+}$, decreases linearly with increasing *k*, however, the $P_{max}^{opt}$ under alternation between two light levels responds in a nonlinear manner with respect to the parameter *k*. The highest rate of change in $P_{max}^{opt}$ is attained for values of *k*<1/(1+α) (for details see the Supplementary Data S1B). It is important to observe that $P_{max}^{opt}$ is larger than ${\bar{P}}_{max}$ for *k*<1/(1+α) indicating that $P_{max}$ must typically be elevated in order to attain an optimized response in fluctuating light conditions.

Next, we analysed how the amplitude of fluctuations influences $P_{max}^{opt}$ by keeping the averaged light intensity constant and setting *k*=1/2, but changing the light intensities *L*_-_ and *L*_+_. We defined intensities as $L_{\pm }=\bar{L}\left(1\pm x\right)$, where 0≤*x*≤1, so that for *x*=1, for example, light intensity switches between zero and $2\bar{L}$. Figures 2C, D show the fluctuating light pattern and $P_{max}^{opt}$ as a function of *x*. In this case, $P_{max}^{opt}$is consistently greater than ${\bar{P}}_{max}$ by an amount that increases with the amplitude of the light fluctuation.

Light in nature is much more heterogeneous and unpredictable than that considered so far. One simple optimisation problem is to consider how to maximize daily carbon gain given that *L* is a fluctuating quantity. Analysis based on a small-amplitude approximation (details of which are given in the Supplementary Data S1) shows how $P_{max}^{opt}$rises in proportion to *x* ^2^ for small values of *x*; this approximation is indicated by the dashed line in Fig. 2D. It captures predictions of the numerically computed $P_{max}^{opt}$ in this example for values of light intensity up to approximately 100 μmol m^–2^ s^–1^.

### Influence of the light intensity switching period

We have considered so far that the leaf reacts to light intensity dynamics in a cumulative manner by determining the fraction of time it has been exposed to various intensities of light. Experimental evidence shows maximum photosynthetic capacity depends on the pattern of switching between high and low light intensity (Yin and Johnson, 2000). To account for this we apply a time-weighted average to the light pattern [see Eq. (3)]. We now consider how the light-switching period influences the optimal photosynthetic rate when the leaf has a fading memory.

We set *k*=1/2, so that *L*(*t*) equals *L*_-_ or *L*_+_ for equal amounts of time in total, but now vary the number of *L*_-_ to *L*_+_ switches within a photoperiod of duration *T*. The switching period, *S,* specifies the time required to have a single continuous low light to continuous high light cycle, so that *S*=*T* indicates no repeats of the light pattern.

**Fig. 3.**
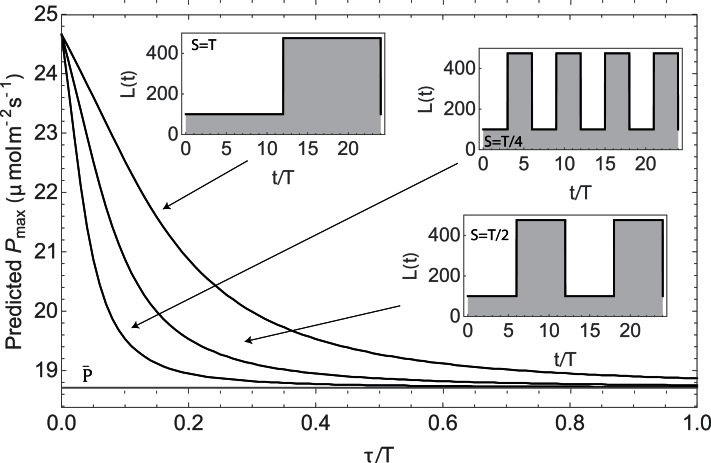
Influence of the light switching period, *S*, and time-weighted average timescale, τ, on $P_{\text{max}}^{\text{opt}}$. Light fluctuated between *L*_-_=100 μmol m^–2^ s^–1^ and *L*_+_=475 μmol m^–2^ s^–1^ for periods *S*=T, *S*=T/2 and *S*=T/4 (black lines). Grey line shows the predicted $P_{max}$ for $\bar{L}$ =287.5 μmol m^–2^ s^–1^.

Figure 3 shows how photosynthetic capacity changes as a function of τ for three fluctuating light patterns with switching period *S*=*T*, *T*/2 and *T*/4, i.e. the low/high light pattern changes one, twice or four times. As τ increases, $P_{max}^{opt}$ decreases steadily until it reaches ${\bar{P}}_{max}$, the optimal value when the light pattern is replaced with its average. For a fixed time-averaging timescale τ, the light patterns with shorter switching periods are closer to $\bar{L}$ after the time averaging than the longer switching periods, making $P_{max}^{opt}$closer to ${\bar{P}}_{max}$.

### Fluctuating light

**Fig. 4.**
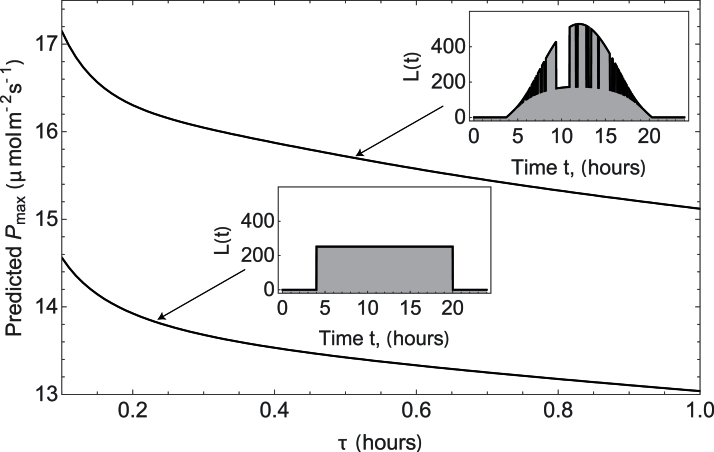
Predicted $P_{max}$ as a function of τ for a typical diurnal variation in PPFD at a particular point inside a canopy and a pattern with a fixed *L*=251.7 μmol m^–2^ s^–1^ over 16h.

As a proof of concept we applied our proposed mathematical framework to a light pattern corresponding to a typical diurnal variation in PPFD at a particular point inside a canopy. The direct component of PPFD fluctuates due to the solar movement and canopy architecture; a detailed pattern of PPFD can be obtained using a direct ray-tracing algorithm (Song *et al.*, 2013). Figure 4 shows a fluctuating light pattern and a pattern with fixed *L*=251.7 μmol m^–2^ s^–1^ over 16h, both having the same integrated PPFD. Again, as we increase value of τ, the $P_{max}^{opt}$ decreases; however, it is higher for fluctuating light compared to the fixed PPFD because of the differing patterns of variation in the light intensity on timescales longer than τ.

### Comparison with experimental data

**Fig. 5.**
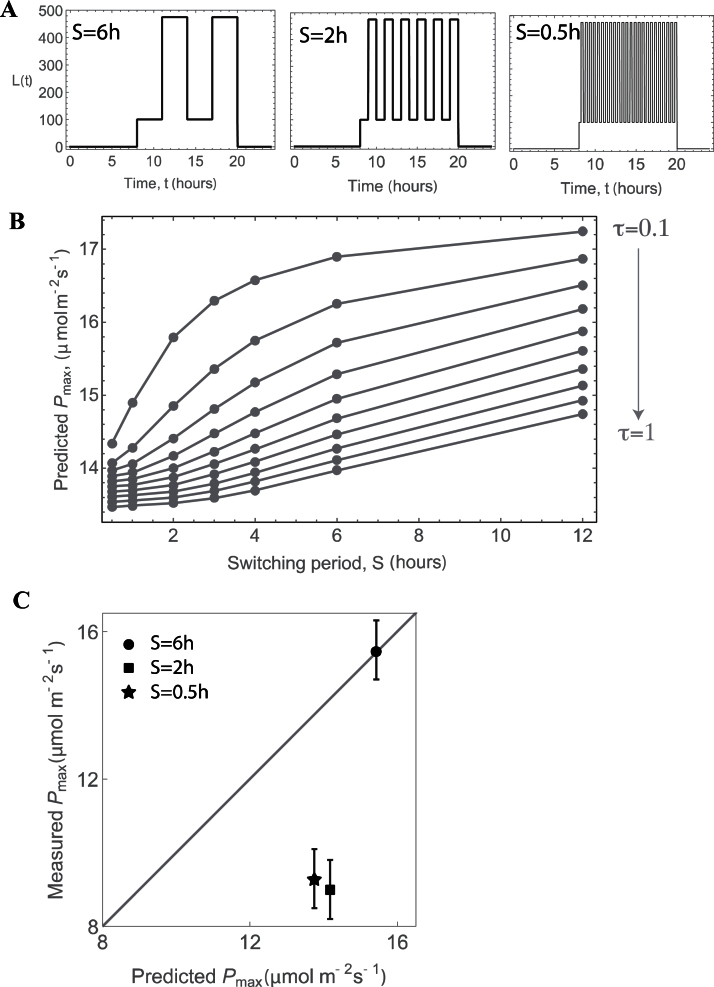
Comparing model predictions with experimental data. (A) Experimental set-up for 0.5h, 2h and 6h PPFD switching. (B) Predicted $P_{max}$ as a function of switching period for τ values from 0.1h to 1h. (C) Comparison between measured and predicted (τ=0.3h) maximum photosynthetic capacity; grey line shows 1:1 values.

Model predictions were calculated for light fluctuating for 12h between 100 and 475 μmol m^–2^ s^–1^ at switching periods *S* = 0.5, 1, 2, 4, 6 and 12h, which correspond respectively to 24, 12, 6, 3, 2 and 1 switches from low to high light. All light patterns have the same integrated PPFD of 12.42mol m^–2^ d^–1^. Figure 5A show light patterns for *S*=0.5, 2 and 6h.

By numerically optimizing daily carbon gain for time-weighted averaged light patterns over 24 hours as given in Eq. (2), we calculated the optimal maximum photosynthetic capacity as a function of *S* for values of τ in the range from 0.1h to 1h with a step of 0.1h (Fig. 5B). We found an inverse relationship between the maximum photosynthetic capacity and the frequency of low to high light transitions.

In Fig. 5C we plotted predicted $P_{max}^{opt}$ versus experimentally measured $P_{max}$ (Yin and Johnson, 2000) for light patterns given in Fig. 5A. With τ=0.3h there is good agreement between experiment and theory for the 6h switching period (RMSE=0.89). Although the model predicts the correct trend in light response curves for *S=*2h and *S=*0.5h, it predicts higher values of $P_{max}$ compared to experimentally measured light response curves. Nevertheless, the model is valuable in providing a mechanistic explanation for the observed general increase in $P_{max}$ with switching period.

## Discussion

We have formulated a mathematical framework of dynamic acclimation in order to define the optimal adjustments to net photosynthesis under fluctuating light conditions. We have found that the effect of different light patterns on maximum photosynthetic capacity has two main features: (i) for a light pattern with two levels of irradiance, the increase in optimal $P_{max}$ depends on the fraction of time under low light versus high light; and (ii) for a light pattern switching between low and high light at different frequencies, optimal $P_{max}$ is greater under a lower frequency of low light and high light transitions. These predictions offer a practical way of assessing whether the acclimation status of any given leaf is best adapted to its dynamic environment. However it is currently difficult to test this model with a broad range of data: the majority of experimental work carried out so far on acclimation has used steady-state conditions that do not reflect natural or agricultural environments accurately.

Previous empirical work showed that ability to undergo dynamic acclimation can affect biomass and fitness (Athanasiou *et al.*, 2010). Similarly, optimization of short-term photoprotective responses to light dynamics can influence fitness (Kulheim *et al.*, 2002). However, regulatory aspects of acclimation and how they adapt under highly variable light patterns are less well understood. This paper represents the first step to addressing this problem. The quasi-steady net photosynthetic rate model we present here offers a clear framework that explains how dynamic acclimation may function in a complex light environment. This approach, where dynamic leaf responses are linked to environmental change in a quantitative manner in order to define optimal responses for productivity, has practical applications. For example there are implications for crop biomass and yield although any improvement would need a firm genetic basis.

Daily carbon gain cannot be derived from the average values of light due to the highly non-linear response of photosynthesis to light (Niinemets and Anten, 2009). Indeed measured profiles of photosynthetic capacity in plant crowns typically do not match those of average irradiance (Buckley *et al.*, 2013). The results of the present study indicate that the optimal maximum photosynthetic capacity under fluctuating light patterns is different compared to those obtained from averaged light intensity. When comparing light patterns with the same integrated and peak PPFD, but with different intensity patterns, we found that the maximum photosynthetic capacity was reduced when the frequency of transitions was increased. This is in agreement with the dynamic acclimation data in existence for *A. thaliana*, grown under light patterns alternating for 12h between 100 μmol m^–2^ s^–1^ and 475 μmol m^–2^ s^–1^ over time periods of 30min, 2h and 6h (Yin and Johnson, 2000). Furthermore, a value of τ ~0.3h broadly agrees with the experimentally observed decrease in $P_{max}$.

We show here that the optimal maximum photosynthetic capacity was higher than that obtained for the averaged light intensity if the fraction of higher light intensity was large enough (Fig. 2B), even under small-amplitude light fluctuations. The relative advantage of $P_{max}^{opt}$ over ${\bar{P}}_{max}$ increased with increasing difference between two levels of irradiance (Fig. 2D). Our study extends early work by Takenaka (1989), which employed a broadly similar mathematical approach but was based on optimal photosynthetic capacity of a leaf maximizing daily carbon gain estimated over an entire leaf lifetime, and found that the relative frequency distribution of irradiance rather than its average was critical when predicting optimal $P_{max}$.

Early notable work by Robert Pearcy and others (Pearcy, 1990) showed how light dynamics in plant canopies can contribute to productivity. The acclimation status of leaves within a canopy determines their ability to utilize light flecks effectively. However there are other factors that are thought to interact with acclimation to determine the final photosynthetic properties of a leaf, for example nitrogen (N) content. A long-established theory of optimal distribution of photosynthetic resources predicts that for a given total canopy N content, there is a 1:1 relationship between $P_{max}$ and the light intensity (Field, 1983). However, experimental data indicates that maximum photosynthetic capacity does not precisely match the light vertical gradient within a canopy (Kull, 2002). One of the assumptions of canopy optimization theory is that the distribution of light absorption among leaves is constant (Foulkes and Murchie, 2011; Niinemets, 2012). But this ratio changes depending on various factors such as time of day, solar elevation and cloud cover (Terashima *et al.*, 2005). Therefore the temporal fluctuations in PPFD should be explicitly considered when establishing the distribution of $P_{max}$ (Posada *et al.*, 2009). To the best of our knowledge, methods for determining the efficiency of light acclimation for given complex patterns of light history have not been proposed in the literature. In addition there may be genetic constraints on the capacity of particular species to acclimate (Anderson *et al.*, 1995; Murchie *et al.*, 1998; Athanasiou *et al.*, 2010). This is the case for shade-adapted or sun-adapted species for example. However empirical knowledge of the optimal photosynthetic response for a given environment will allow acclimation to light to finally be placed in proper context, with limitations placed by other biotic and abiotic factors.

Previously, the acclimation status of leaves within a given plant canopy has been compared to average light level (Niinemets and Anten, 2009). We can now test the hypothesis that it is defined by the dynamic properties of the canopy and discover the limitations placed by other biological properties such as nitrogen remobilization dynamics discussed above. With knowledge of canopy architecture, we can define the pattern of light via ray-tracing algorithms such as those used by Song *et al.* (2013) and calculate the predicted light history for canopy positions and layers. We applied our proposed mathematical framework to a typical diurnal variation in PPFD at a particular point inside a canopy (Fig. 4). However it is first necessary to verify predictions of an optimal $P_{max}$ under a realistic variation in light environment and this requires experiments conducted under controlled conditions with precisely regulated complex light patterns and appropriate photosynthesis measurements. The final verification will arise from field testing.

There are very few experimental investigations producing data that would allow us to understand the influences of light pattern on dynamic acclimation. This may be partly due to past difficulties in developing lighting systems that could cope with rapid switching between light levels of greatly differing magnitude. Recent developments with LED lighting have overcome such problems and it is now possible to accurately replicate light dynamics from virtually any environment. The model we present here should be considered a tool for the analysis of optimal leaf acclimation to variable light environments.

We have considered one factor, light energy input, and we view this method as a basis for more complex assessments that would parameterize the model with data affecting photosynthesis *in situ* such as leaf temperature, humidity and nutrients. We have not incorporated photosynthetic induction, i.e. the overall relative induction state (Stegemann *et al.*, 1999; Kuppers and Pfiz, 2009), into our model; instead we introduced a time-weighted average for the light pattern. A model that incorporates induction would need to be supported by data from light-fleck acclimation experiments. Full parameterization of such a model will require high-resolution measurements of a time course of PPFD, as well as photosynthesis rates.

Another aspect, which will require future experimental data and model testing, is the inhibition of R_d_ in the light. For a given light intensity and temperature the level of inhibition is reasonably constant between species (Atkin *et al.*, 1997). Whether there is a variation in the level of inhibition according to the growth light treatment is in itself is an interesting point.

## Conclusions

Acclimation, sometimes referred to as plasticity, is an essential component of environmental adaptation but assessment of ‘effectiveness’ in a complex temporal and spatial environment can be difficult. There is a need to determine how efficiently leaves utilize light for photosynthesis in fluctuating conditions for ecological understanding and agricultural improvements. Our straightforward approach to develop a model for determining the efficiency of light acclimation in a given environment is a significant step forward and we propose it as the basis for a new physiological tool. We show that it is possible to take into account complex patterns of light history, the behaviour of processes such as induction state and for such a model to be consistent with available data. We anticipate that future experimental investigations will produce data necessary for further validation and refinement of the model.

## Supplementary Data

Supplementary data are available at *JXB* online.

Supplementary Data S1. (A) Quasi-steady net photosynthetic rate model. (B) Analysis of light intensity regime under alternation between two light levels. (C) Small amplitude light fluctuations.

Supplementary Data
