# Supplementary material for: Exploiting heterogeneous environments: does photosynthetic acclimation optimize carbon gain in fluctuating light?
Source: J Exp Bot. 2015 Mar 18;66(9):2437–47. doi: 10.1093/jxb/erv055 (PMC4629418; doi:10.1093/jxb/erv055)
Supplement: Supplementary Data [file supp_66_9_2437__index.html]

Exploiting heterogeneous environments: does photosynthetic acclimation optimize carbon gain in fluctuating light? — Exploiting heterogeneous environments: does photosynthetic acclimation optimize carbon gain in fluctuating light? — Supplementary Data 

# Exploiting heterogeneous environments: does photosynthetic acclimation optimize carbon gain in fluctuating light?

## Supplementary Data

Data files

**Files in this Data Supplement:**

- Supplementary Data - Supplementary Data
