# Supplementary material for: Exploiting heterogeneous environments: does photosynthetic acclimation optimize carbon gain in fluctuating light?
Source: J Exp Bot. 2015 Mar 18;66(9):2437–47. doi: 10.1093/jxb/erv055 (PMC4629418; doi:10.1093/jxb/erv055)
Supplement: Supplementary Data [file supp_erv055_jexbot143016_file001.pdf]

Supplementary material

Exploiting heterogeneous environments: does photosynthetic acclimation optimise carbon gain in fluctuating light?

R. Retkute, S. Unna, R. Smith, A.J. Burgess, O.E. Jensen, G.N. Johnson, S.P. Preston, E.H. Murchie

August 31, 2014

A Quasi-steady net photosynthetic rate model

The relationship between the net photosynthetic rate P and light intensity L (Fig. 1) possesses the following properties: $P \to P_{\max}$ for large L (saturation); $P(0, P_{max}) = R_D < 0$ where $-R_D = \alpha P_{\max}$ for some $\alpha > 0$; $P(L_c, P_{max}) = 0$, where L_c is the light compensation point; and $\frac{\partial P}{\partial L}(L_c, P_{max}) = \phi$. In this study, baseline parameters are $\alpha = 0.1$ and $\phi = 0.0124$ mol CO_2 per mol photons.

Two simple models for P versus L are

$$P^{(1)}(L, P_{\max}) = \begin{cases} -\alpha P_{\max} + \phi L & \text{if } 0 < L \le \frac{(1+\alpha)P_{\max}}{\phi}, \\ P_{\max} & \text{if } L > \frac{(1+\alpha)P_{\max}}{\phi}, \end{cases} \tag{1a}$$

$$P^{(3)}(L, P_{\max}) = \frac{\phi L + (1+\alpha)P_{\max} - \sqrt{(\phi L + (1+\alpha)P_{\max})^2 - 4\theta\phi(1+\alpha)LP_{\max}}}{2\theta} - \alpha P_{\max}. \tag{1b}$$

The parameter θ determines the curvature of the response curve (1b). For $\theta = 1$, (1b) becomes $P^{(3)}(L, P_{\max}) = \frac{1}{2}\left(P_{\max}(1-\alpha) + L\phi - |P_{\max}(1+\alpha) - L\phi|\right)$, which is equivalent to (1a).

The value of $P_{\max}$, which we denote $P^*_{\max}$, that maximises P for given L satisfies

$$P^{*(1)}_{\max}(L) = \frac{\phi L}{1+\alpha}; \tag{2a}$$

$$P^{*(3)}_{\max}(L) = \frac{L\phi\left(\alpha\left(-1+\alpha\left(\theta-1\right)\right)\left(2\theta-1\right) - \left(1+\alpha-2\alpha\theta\right)\sqrt{\alpha\left(-1+\alpha\left(\theta-1\right)\right)\left(\theta-1\right)}\right)}{\alpha\left(1+\alpha\right)\left(-1+\alpha\left(\theta-1\right)\right)}. \tag{2b}$$

Here $\frac{\partial P}{\partial P_{\max}} = 0$ defines $P^*_{\max}$ in (2b); (2a) is then determined from the limit $\theta \to 1$.

B Light intensity regime under alternation between two light levels

Suppose that the light intensity $L(t)$ switches with period T between two different intensities $L_\pm$, where $L_- \le L_+$. For $0 \le t \le kT$, $L(t) = L_-$, where $0 \le k \le 1$; $L(t) = L_+$ otherwise.

The net carbon assimilation rate for such an $L(t)$ is

$$C(k, L_-, L_+) = \frac{1}{T}\int_0^{kT} P\left(L_-, P_{\max}\right) \mathrm{d}t + \frac{1}{T}\int_0^{(1-k)T} P\left(L_+, P_{\max}\right) \mathrm{d}t, \tag{3}$$

giving

$$C^{(1)}(k, L_-, L_+) = \begin{cases} -\alpha P_{\max} + \phi\left(kL_- + (1-k)\,L_+\right), & \text{if } L_+ \le \frac{(1+\alpha)P_{\max}}{\phi}, \\ k\left(-\alpha P_{\max} + \phi L_-\right) + (1-k)\,P_{\max}, & \text{if } L_- \le \frac{(1+\alpha)P_{\max}}{\phi} < L_+, \\ P_{\max}, & \text{if } \frac{(1+\alpha)P_{\max}}{\phi} < L_-, \end{cases} \tag{4a}$$

$$\begin{aligned} C^{(3)}(k, L_-, L_+) = {}& k\frac{L_-\phi - \sqrt{-4L_-P_{\max}(1+\alpha)\theta\phi + \left(P_{\max}(1+\alpha) + L_-\phi\right)^2}}{2\theta} \\ & + (1-k)\frac{L_+\phi - \sqrt{-4L_+P_{\max}(1+\alpha)\theta\phi + \left(P_{\max}(1+\alpha) + L_-\phi\right)^2}}{2\theta} \\ & - P_{\max}\alpha + \frac{P_{\max}(1+\alpha)}{2\theta}. \end{aligned} \tag{4b}$$

According to (4a), the maximum net carbon assimilation rate is attained for $P_{\max} = \phi L_\pm/(1+\alpha)$ (depending on the value of k), with the transition between the two states arising for $\partial C/\partial P_{\max} = 0$, with

$$k = \frac{1}{1+\alpha}. \tag{5}$$

Thus for $0 < k < 1/(1+\alpha)$, $P_{\max}^{opt} = P_{max}^*(L_-) > \bar{P}_{max}$, whereas for $1/(1+\alpha) < k < 1$, $P_{\max}^{opt} = P_{max}^*(L_+) < \bar{P}_{max}$. This step-like property remains evident in Figure 3, for which $\theta = 0.99$, although in this case $P_{\max}^{opt}$ is typically larger then $\bar{P}_{max}$, indicating that P_{max} must be elevated in order to attain an optimised response in fluctuating light conditions.

C Small amplitude light fluctuations

Light in nature is much more heterogeneous and unpredictable than that considered in previous sections. Here we consider how to maximise C when L is a fluctuating quantity. Specifically, suppose that $L = \overline{L} + \hat{L}$, where the bar denotes a time average and by definition $\overline{\hat{L}} = 0$. If the fluctuations are infinitesimal, we anticipate that C is maximised by $\overline{P}_{\max} = P_{max}^*(\overline{L})$. We now determine how $P_{\max}^{opt}$ must change in order to accommodate the fluctuations. We write the change as the constant $\hat{P}_{\max}$. For clarity of notation we express the response curve as $P = F(L, P_{\max})$ for some function F.

Setting $P_{\max} = \overline{P}_{\max} + \hat{P}_{\max}$, where $\hat{P}_{\max}$ is to be determined, we Taylor expand F, treating $\hat{P}_{\max}$ as being of comparable magnitude to $\hat{L}^2$:

$$P = F(\overline{L}, \overline{P}_{\max}) + F_L(\overline{L}, \overline{P}_{\max})\hat{L} + F_P(\overline{L}, \overline{P}_{\max})\hat{P}_{\max} + \tfrac{1}{2}F_{LL}(\overline{L}, \overline{P}_{\max})\hat{L}^2 + \dots \tag{6}$$

where $F_P \equiv \partial F/\partial P_{\max}$. Evaluating C for large T, so that C is the time-average of P, it follows that

$$C = F + F_P\hat{P}_{\max} + \tfrac{1}{2}F_{LL}\overline{\hat{L}^2} + \dots. \tag{7}$$

Maximising C over $P_{\max}$ requires $C_P = 0$, i.e. $F_P + F_{PP}\hat{P}_{\max} + \frac{1}{2}F_{PLL}\overline{\hat{L}^2} + \dots = 0$. However derivatives are evaluated at $L = \overline{L}$, $P_{\max} = \overline{P}_{\max}$ and $F_P(\overline{L}, \overline{P}_{\max}) = 0$. implying that

$$\hat{P}_{\max} = -\overline{\hat{L}^2}\frac{F_{PLL}}{2F_{PP}}, \quad (F_{PP} \neq 0). \tag{8}$$

For the light saturation model given by (1b), (8) gives

$$P_{max}^{opt} = \overline{P}_{max} + \frac{\overline{\hat{L}^2}}{2}\frac{\overline{P}_{max}\left(\overline{P}_{max}^2(1+\alpha)^2 + \overline{L}\overline{P}_{max}(1+\alpha)(2\theta-1)\phi - 2\overline{L}^2\phi^2\right)}{\overline{L}^2\left(\overline{P}_{max}^2(1+\alpha)^2 - 2\overline{L}\overline{P}_{max}(1+\alpha)(2\theta-1)\phi + \overline{L}^2\phi^2\right)}. \tag{9}$$

Numerical calculations over range of parameter values show that second term in the above expression is positive, therefore $P_{max}^{opt} > \overline{P}_{max}$ if $\overline{\hat{L}^2} > 0$. This is illustrated in Fig. 4. Thus $P_{\max}^{opt}$ must increase in the presence of fluctations as a consequence of the shape of the underlying light-response surface.
